# Effect of Oral PUVAsol on the Quality of Life in Indian Patients Having Chronic Plaque Psoriasis

**DOI:** 10.1155/2014/291586

**Published:** 2014-09-04

**Authors:** Pratik Gahalaut, Nitin Mishra, Puneet S. Soodan, Madhur K. Rastogi

**Affiliations:** Department of Dermatology, Shri Ram Murti Smarak Institute of Medical Sciences, Bareilly 243202, India

## Abstract

*Background*. Psoriasis is associated with a high impact on health-related QoL (quality of life). PUVAsol has been successfully used for treating psoriasis instead of standard PUVA therapy in developing countries. However, data for PUVAsol therapy and its effect on QoL in psoriatic patients is meagre. *Objective*. To investigate the effect of PUVAsol on the quality of life in patients having chronic plaque psoriasis. *Materials and Methods*. An observational prospective study done in patients having chronic plaque psoriasis. PASI and DLQI were calculated before initiating treatment with oral PUVAsol. These were compared with the respective scores after 12 weeks of regular treatment with PUVAsol. Statistical analysis was done using SPSS version 20.0. *Results*. Both PASI and DLQI showed statistically significant reduction after 12 weeks of regular treatment. 90% of patients responded favourably to PUVAsol therapy in the study and all the domains of DLQI showed significant reduction except domain of “work and school.” *Conclusion*. Our results show that regular PUVAsol treatment improves the physical appearance of disease as evident by decrease in PASI scores. It also improves the QoL of the patients. This study will add upon the growing evidence of efficacy of PUVAsol.

## 1. Introduction

Psoriasis is a common, chronic, inflammatory, and proliferative condition of the skin [1]. In India, the prevalence of psoriasis varies from 0.44 to 2.8% [2]. There is evidence that psoriasis is associated with a high impact on health-related QoL (quality of life) [2]. For a psoriatic patient, measures of morbidity have a far greater relevance compared to mortality. It is currently accepted that the evaluation of disease severity should include clinical, psychological, and social factors [3]. QoL assessment has become an important endpoint in clinical trials in addition to the traditional clinical outcomes [4]. In developing countries QoL issues have not yet gained popularity due to lack of awareness among workers in health sector [4]. Any treatment of psoriasis should be considered ineffective until it improves QoL in patients. Patients with moderate to severe psoriasis generally require phototherapy (e.g., narrowband ultraviolet B radiation), photochemotherapy (oral psoralen plus ultraviolet A radiation), or systemic agents (e.g., cyclosporine, methotrexate, oral retinoids, and fumaric acid esters) to control their disease adequately [5]. Photochemotherapy (PUVA) is the combined use of the drug psoralen and UVA (ultra violet A) radiation to achieve an effect not achieved with the individual components alone [6]. PUVAsol is the intake of psoralen followed by sun exposure as a source of UVA [6, 7]. PUVAsol has been used successfully for treating psoriasis [7–10]. Though a wealth of international data is available regarding QoL in psoriasis, data for PUVAsol therapy and its effect on QoL in psoriatic patients is meagre [4, 11]. There is no data in the literature regarding changes in the quality of life in terms of dermatology life quality index (DLQI) for patients of psoriasis after PUVAsol therapy. Hence this study was designed primarily to measure the effect of PUVAsol therapy on QoL in patients having psoriasis.

## 2. Objective

Primary objective of the study was to investigate the effect of PUVAsol on the quality of life in patients having chronic plaque psoriasis.

## 3. Materials and Methods

This study was done in the Department of Dermatology at Shri Ram Murti Smarak Institute of Medical Sciences, Bareilly (India), from January 2012 to June 2013. All the patients presenting in psoriasis clinic were screened and enrolled in the study based on below mentioned inclusion and exclusion criteria. Inclusion criteria were patients of chronic plaque psoriasis; aged ≥18 years; having >10% body surface area involvement; literate; diagnosed with psoriasis for ≥3 months; willing for treatment, inclusion in study, and regular follow-up. Patients with hepatic or renal impairments, photodermatoses, past or present history of any malignancy or immunobullous disorder, any chronic systemic disorder, and pustular or erythrodermic psoriasis, patients having psoriatic arthropathy and concurrent administration of any phototoxic drugs, and patients who took treatment irregularly, chronic alcoholics and/or smokers and pregnant or lactating females were excluded from the study. A washout period of 2 and 4 weeks was given for topical and systemic therapies, respectively, before including the patients in study.

Written informed consent from all the subjects was taken before recruitment in study. Ethical review committee of our institution approved the study. History, examination, baseline PASI (psoriasis area severity index) score, and relevant investigations were recorded in a specially designed proforma. Patients were also asked to fill a validated Hindi version of DLQI (Dermatology Life Quality Index) questionnaire [12]. PASI and DLQI scores were assessed by a single investigator in the present study. The PASI and the DLQI are the most cited and most often used tools for experimental and descriptive studies due to their high degree of reliability, applicability, and reproducibility [13].

PASI is a physician assessed score. It is recognized by the USA Food and Drug Administration to assess the efficacy of psoriasis therapies in clinical trials. It takes into account the extent of involved skin surface area and severity of erythema, desquamation, and plaque induration [14]. DLQI is a self-administered, easy and user-friendly, dermatology-specific quality of life instrument/questionnaire with an average completion time of 126 s [4]. The total DLQI ranges between 0 (no impairment) and 30 (maximum impairment). The 10 questions in the DLQI can be subdivided into six domains that relate to different aspects of a person's health-related QoL as follows: symptoms and feelings (questions 1, 2), daily activities [3, 4], leisure [5, 6], work/school [7], personal relationships [8, 9], and treatment [10]. Higher scores mean greater impairment of the patient's QoL and vice versa [4].

The patients were counselled about the duration of treatment, the need for regular followup, and probable side effects that could be encountered during treatment. Patients were then started on oral PUVAsol therapy. In the absence of a standard protocol for PUVAsol, the most frequently followed protocol was selected which is described below [15]. For oral PUVAsol, 8-methoxypsoralen (8-MOP) was administered orally in morning with breakfast followed by sunlight exposure, after an interval of 2 hours on 3 alternate days in a week. The 8-methoxypsoralen (8-MOP) was administered at a fixed dose of 0.6 mg/kg. The sunlight exposure was for 5 minutes initially, preferably between 11 a.m. and 3 p.m., and then exposure time was increased by 5 minutes to a maximum of 30 minutes at every alternate subsequent exposure depending on side effects. We could not calculate the minimal phototoxicity dose (MPD) of patients due to financial constraints. Hence it was pertinent to start PUVAsol for the minimal acceptable time period to avoid side effects. Though there is no standard protocol for treating patients with PUVAsol, authors selected the most frequently followed schedule which recommends initial exposure time limit of 5 minutes [16]. PUVAsol exposure daytime limit was chosen based on the above mentioned protocol because solar ultraviolet irradiation is maximum in midnoon. In the past Balasaraswathy et al. measured UVA and UVB irradiance in India and recommended that ideal time for PUVAsol should be between 9:30 a.m. and 3:30 p.m. [17]. Eye protection with UVA blocking glasses was requested from the time of ingestion of psoralen until sunset on the day of exposure. The standard topical therapy was emollients in the form of light liquid paraffin only. End point of treatment was completion of 12 weeks of regular treatment. DLQI and PASI were again assessed at the end of 12 weeks of regular treatment and compared with the respective baseline scores. Hepatic and renal function tests were done at baseline and then repeated at an interval of 4 weeks till the end of study time period.

Statistical analysis was done using SPSS version 20.0. Paired and unpaired* t*-tests were used for comparing the DLQI and PASI scores and results are expressed as mean ± SD.* P* value of <.05 at a CI of 95% was taken as statistically significant.

## 4. Results

**Figure 1:**
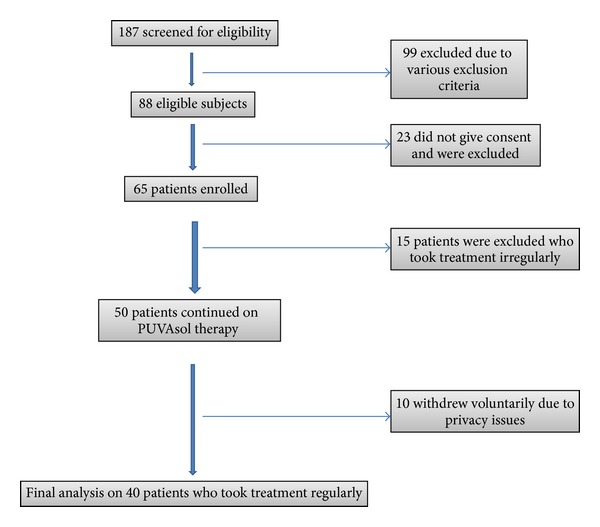
Schematic flowchart of study subjects.

Altogether, 187 patients were screened and only 88 patients were deemed fit to be enrolled in the study due to various inclusion/exclusion criteria. However, only 65 patients gave consent for enrolment in the study. Out of these 65, only 40 patients completed the study. 15 patients took the treatment irregularly and hence were excluded from the study. Another 10 patients withdrew voluntarily due to privacy issues as they had difficulty in exposing their bodies to sunlight. Hence final analysis was done on 40 patients (Figure 1).

Table 1 describes the demographic and clinical characteristics of study patients. All our patients had Fitzpatrick skin phototype IV. To assess the efficacy of PUVAsol, patients in the study groups were classified depending on the site of lesions on their body into different subgroups, namely, exposed (lesions only on exposed parts), unexposed (patients having lesions on unexposed parts only), and mixed (lesions present on both exposed and unexposed parts of the body). However, none of the patients had lesions in only exposed parts. Subsequently the patients were divided into 2 subgroups: those having lesions at the unexposed sites only and others having lesions at mixed sites. PASI scores in different subgroups of study patients have been described in Table 2.  Table 3 describes the DLQI scores in study patients.

90% (36/40) of patients responded to the treatment and achieved reduction in PASI scores after 12 weeks of regular PUVASOL. While 20% of patients (8/40) achieved ≥75% reduction from baseline PASI scores, 40% (16/40) of patients had 50–74% reduction, 10% (4/40) of patients showed 40–49% reduction in PASI scores, and 20% (8/40) of patients achieved 30–39% reduction in baseline PASI scores after 12 weeks of regular treatment. Rest 10% (4/40) of the patients reported worsening of the condition, that is, increase in PASI, even after regular treatment.

As per Table 4, after regular treatment with PUVASOL for 12 weeks, all the domains of DLQI scores showed statistically significant reduction except the domain of “work and school.” It is noteworthy that the domain of DLQI (symptom and feeling) having highest score at baseline showed highly significant reduction after regular treatment.

During haematological investigations no hepatic or renal impairment was detected in the study patients. 28 patients (70%) experienced side effects. These were nausea in 26/40 (65%), hyperpigmentation in 12/40 (30%), headache in 8/40 (20%), pruritus in 4/40 (10%), and phototoxicity in 4/40 (10%) of study patients. However, none of the patients withdrew from the study due to these side effects.

## 5. Discussion

PUVA has a beneficial effect in psoriasis and other skin diseases [6]. While artificial UV radiation, which allows precise dosing, has been available for last few decades, the recognition of the therapeutic effect of sunlight, of which UV light comprises a proportion, goes back to ancient times [6]. Though, the controlled irradiance of an artificial light source is preferable, 8-methoxypsoralen in conjunction with sunlight exposure is also effective [18]. In a tropical country like India, sun is an inexpensive and inexhaustible source of UVA almost throughout the year. PUVAsol is the most commonly used mode of phototherapy for treating psoriasis in India as artificial chambers for photochemotherapy are not readily available [6]. PUVASOL does not require a costly set-up, can be administered at home, and has better compliance as compared with PUVA [6, 7]. Moreover, clinical efficacy of PUVAsol is comparable to PUVA and PUVAsol has a favourable cost effectiveness ratio [7].

In the present study, males constituted the majority of patients. This is in concurrence with most of the Indian studies which have reported a higher prevalence of psoriasis in males [9, 10, 19–23]. It can be attributed to the fact that the male patients come forward for examination and treatment. On the other hand, there is hesitancy on the part of females to come forward for treatment, which may be due to fear of social stigma and/or rejection.

Mean baseline PASI score of 22.94 in the present study is much more than an earlier Indian study describing the clinical efficacy of PUVAsol [7]. The difference may be due to the varied severity of patients included in these studies. Besides PASI is a semiquantitative and subjective score with limited interrater reliability [24].

90% of the patients responded to PUVAsol therapy in the present study. This is more than the response seen in past studies [7, 9, 10]. Recently Aggarwal et al. reported positive response in 75% of psoriatics after 12 weeks of PUVAsol therapy [7]. Kar et al. and Talwarkar et al. reported marked improvement in 44% of patients and >50% improvement in 63% of patients, respectively, with PUVAsol [9, 10]. The difference in response rate may be attributable to the different study period in past studies or the difference in the baseline severity of psoriasis among patients included in the above mentioned studies. Further, quantification of ultraviolet light in PUVAsol depends on the season, time of the day, latitude, conditions of the atmosphere, and time of exposure [18].

In the present study, the decrease in PASI scores was not statistically significant among patients having lesions on mixed sites. This may be due to the small sample size and higher baseline PASI score in these patients compared to patients having lesions on only unexposed sites.

In our study, 24/40 (60%) of study subjects achieved at least ≥50% of improvement in PASI scores after 12 weeks of regular treatment. Marquis and Rangwala reported marked improvement in 79.2% of patients in 8–12 weeks with PUVAsol [8]. In a recent study 65% of patients of chronic plaque psoriasis achieved PASI 90 within 8 weeks [7].

Although sunlight is largely beneficial, in a small minority of patients psoriasis may be provoked by strong sunlight and cause summer exacerbations in exposed skin [25]. This may be a possible explanation for the worsening of disease in 10% of patients after PUVAsol therapy.

The baseline psoriasis severity of patients included in the present study, in terms of mean DLQI, was 14.45. This is comparable to mean DLQI scores of 10.6 to 18.83 reported among psoriatic patients in various past studies done worldwide [11, 26]. Finlay et al. have proposed a banding system to felicitate the clinical interpretation of DLQI scores [4, 27]. The baseline DLQI scores indicate that the patients who presented for treatment in the present study had “very large effect” on overall health-related quality of life (HRQoL) [4].

Many clinical trials have demonstrated the ability of DLQI to detect changes in patients' QoL before and after treatment [4]. In the present study, after 12 weeks of regular treatment, the mean DLQI score reduced to 9.40. In other words, after 12 weeks of treatment, there was statistically significant improvement and a favourable band shift in the DLQI scores from “very large effect” to “moderate effect” [4]. The difference in the mean values of DLQI before and after treatment is clinically meaningful according to the proposed minimal clinical important difference (MCID) of 3.2 for DLQI in psoriasis [28]. Patient reported outcomes, based on DLQI scores, are more sensitive to treatment and precede clinical outcomes in psoriasis [14]. Recent studies have stated that improvement in DLQI paralleled the changes in PASI scores [14].

Interestingly present study also demonstrates that a reduction in PASI of as low as 50% may also translate into significant improvement of QoL in patients treated with PUVAsol. This is in sharp contrast to various earlier studies for psoriasis where endpoint of PASI 75 translated into significant QoL improvement [28]. However, Carlin et al. reported earlier that 50% to <75% improvement in PASI score is associated with improvement in QoL scores and therefore it is a clinically meaningful degree of improvement [29]. This implies that PUVAsol is indeed an effective treatment for psoriasis.

DLQI scores in both the subgroups of patients were comparable at baseline (*P* = 0.9571) and after treatment (*P* = 0.1747). In a past study from developing nation, there was no significant difference in quality of life among patients having either localized or disseminated lesions [30]. After treatment, DLQI scores decreased significantly only in patients belonging to mixed group. Hence patients having lesions on both exposed and unexposed parts of the body had a greater and significant improvement in QoL after 12 weeks of regular PUVAsol therapy. Facial involvement and widespread disease in psoriasis is associated with greater impact of disease on QoL [31]. In the present study also, a relatively lesser improvement in widespread psoriatic lesions and in psoriatic lesions on the exposed parts of the body transcended into a much greater improvement in QoL.

DLQI and PASI scores were compared in the 2 subgroups of patients, depending on the site of lesion. Though the patients having lesions on the unexposed parts had significant reduction in PASI during study period, statistically significant decrease in DLQI score was observed in the other subgroup of patients, which had lesions on mixed sites. DLQI provides a multidimensional view of the effect of disease and treatment. It thereby enables assessment of treatment benefit beyond that demonstrated by clinical measures alone [14]. To identify the areas that were most influenced by the treatment, the DLQI scores were divided into six domains as explained above. The greatest pre/posttreatment difference in DLQI was seen in “symptoms and feelings,” followed by “personal relationships,” “treatment,” “leisure,” and “daily activities.” There are a couple of past studies which have reported similar strong impairments in the domain of “symptoms and feelings” [11, 32]. The variation in total DLQI scores in the present and earlier studies may be a reflection of the differences in geography and cultural practices.

The domain of “work and school” failed to show any statistically significant fall in DLQI scores in the present study. Since our medical college is located in a suburban locality, majority of patients presenting in our department are from lower socioeconomic status and hail from a rural background (Table 1). Occupation of such patients may not be affected by the presence or absence of psoriasis. On the other hand, perceptions of relatives and coworkers towards psoriasis may have a much greater impact on such patients. This perception may be reflected as high scores for the domain of “symptoms and feelings” on QoL index in our study. A recent Brazilian study stated that psoriatic patients having occupation, which involved interaction with familiar or restricted groups of people (retired and rural workers and housekeepers), failed to show any correlation between PASI and DLQ-Bra (Brazilian version of DLQI) both before and after treatment [13].

## 6. Limitations

In our study design, we had no control group and therefore we cannot draw any conclusions about the efficacy of PUVAsol treatment compared to other modalities of treatment for psoriasis. Further a small sample size is another limitation of the present study. It is possible that the patients who were excluded from the study, due to irregular treatment, may have showed lesser or no response. Besides only one disease-specific instrument, that is, DLQI, was used to measure HRQoL. Doubts have been raised in the past regarding the inadequate measurement properties of DLQI [33]. Still, these shortcomings cannot negate the results of the present study.

## 7. Conclusion

The present study shows that PUVAsol has a definitive role in improving QoL in patients having chronic plaque psoriasis. This study will add upon the growing evidence for utility of PUVAsol as well as DLQI in daily practice. This should help dermatologists in a developing country or resource poor settings to make a better and informed decision to promote PUVAsol as a fruitful, convenient, and effective therapy for managing psoriasis. However, large randomized trials are needed to substantiate the results of the present study as the quest for an ideal treatment of psoriasis seems everlasting.

## Figures and Tables

**Table 1:** Demographic and baseline clinical characteristics.

Variables	Study group (*n* = 40)
Age in years (mean) (range)	40.55 (21–70)
Sex ratio (M : F)	4 : 1
Duration in years (mean) (range)	3.98 (1–10)
Family history of psoriasis (%)	16 (40%)
Body site of affliction	
Exposed only	0
Nonexposed only	32 (80%)
Mixed	8 (20%)
Previous treatment taken	
Topical only	4 (10%)
Systemic only	4 (10%)
Both topical and systemic	32 (80%)
Occupation	
Housewife	6 (15%)
Farmer	14 (35%)
Labourer	10 (25%)
Student	4 (10%)
Business	6 (15%)
Household background	
Urban	12 (30%)
Rural	28 (70%)

**Table 2:** PASI scores in study group at baseline and after 12 weeks of regular treatment.

Study subjects	Pretreatment (baseline)	Posttreatment (after 12 weeks)	*P* value
Total cases (*n* = 40)	22.94 ± 9.03	14.16 ± 10.99	0.0002
Patients having lesions at mixed sites (*n* = 8)	29.38 ± 7.30	24.20 ± 16.09	0.0780
Patients having lesions at unexposed sites (*n* = 32)	19.79 ± 7.03	10.81 ± 5.64	0.0001

**Table 3:** DLQI scores in study group at baseline and after 12 weeks of regular treatment.

Type of study subjects	Pretreatment (baseline)	Posttreatment (after 12 weeks)	*P* value
Total cases (*n* = 40)	14.45 ± 3.19	9.40 ± 6.52	0.0004
Patients having lesions at mixed sites (*n* = 8)	14.27 ± 2.66	8.33 ± 4.87	0.0001
Patients with lesions at unexposed sites (*n* = 32)	14.20 ± 5.01	11.20 ± 7.73	0.0587
Patients who achieved ≥75% PASI improvement (*n* = 8)	12.25 ± 3.06	4.00 ± 2.73	0.0001
Patients who achieved 50–75% PASI improvement (*n* = 16)	13.38 ± 2.83	5.75 ± 1.24	0.0001
Patients with <50% PASI improvement (*n* = 12)	16.5 ± 2.39	14.67 ± 4.41	0.1737
Patients reporting worsening of disease (*n* = 4)	16.00 ± 2.31	19.75 ± 2.63	0.0006

**Table 4:** Comparison of different domains of DLQI at baseline and after 12 weeks of treatment.

Domain	DLQI (mean ± SD) before treatment	DLQI (mean ± SD) after treatment	*P* value
Symptoms and feelings	4.20 ± 1.20	2.75 ± 1.62	0.0003
Daily activities	3.10 ± 0.91	2.30 ± 1.81	0.04
Leisure	2.25 ± 0.72	1.45 ± 0.89	0.003
Work and school	1.45 ± 0.69	1.00 ± 1.21	0.07
Personal relationships	2.10 ± 1.55	1.45 ± 1.50	0.0004
Treatment	1.25 ± 0.55	0.45 ± 0.51	0.0004
